# Interactive Effects of Ceftriaxone and Chitosan Immobilization on the Production of Arachidonic Acid by and the Microbiome of the Chlorophyte *Lobosphaera* sp. IPPAS C-2047

**DOI:** 10.3390/ijms241310988

**Published:** 2023-07-01

**Authors:** Svetlana Vasilieva, Alexandr Lukyanov, Christina Antipova, Timofei Grigoriev, Elena Lobakova, Olga Chivkunova, Pavel Scherbakov, Petr Zaytsev, Olga Gorelova, Tatiana Fedorenko, Dmitry Kochkin, Alexei Solovchenko

**Affiliations:** 1 Faculty of Biology, Lomonosov Moscow State University, 1-12 Leninskie Gory, 119234 Moscow, Russia; vankat2009@mail.ru (S.V.); loockart@mail.ru (A.L.); elena.lobakova@gmail.com (E.L.); olga.chivkunova@mail.ru (O.C.); cyano@mail.ru (P.S.); zaytsevpa@my.msu.ru (P.Z.); ogo439@mail.ru (O.G.); tatfed@mail.ru (T.F.); dmitry-kochkin@mail.ru (D.K.); 2 Institute of Natural Sciences, Derzhavin Tambov State University, Komsomolskaya Square 5, 392008 Tambov, Russia; 3 Laboratory of Polymeric Materials, National Research Center “Kurchatov Institute”, Kurchatov Square 1, 123098 Moscow, Russia; kris444ti@yandex.ru (C.A.); timgrigo@yandex.ru (T.G.); 4 Timiryazev Institute of Plant Physiology, Russian Academy of Sciences, Botanicheskaya St. 35, 127276 Moscow, Russia

**Keywords:** *Lobosphaera*, attached cultivation, antibiotics, bioremoval, arachidonic acid

## Abstract

Pharmaceuticals including antibiotics are among the hazardous micropollutants (HMP) of the environment. Incomplete degradation of the HMP leads to their persistence in water bodies causing a plethora of deleterious effects. Conventional wastewater treatment cannot remove HMP completely and a promising alternative comprises biotechnologies based on microalgae. The use of immobilized microalgae in environmental biotechnology is advantageous since immobilized cultures allow the recycling of the microalgal cells, support higher cell densities, and boost tolerance of microalgae to stresses including HMP. Here, we report on a comparative study of HMP (exemplified by the antibiotic ceftriaxone, CTA) removal by suspended and chitosan-immobilized cells of *Lobosphaera* sp. IPPAS C-2047 in flasks and in a column bioreactor. The removal of CTA added in the concentration of 20 mg/L was as high as 65% (in the flasks) or 85% (in the bioreactor). The adsorption on the carrier and abiotic oxidation were the main processes contributing 65–70% to the total CTA removal, while both suspended and immobilized cells took up 25–30% of CTA. Neither the immobilization nor CTA affected the accumulation of arachidonic acid (ARA) by *Lobosphaera* sp. during bioreactor tests but the subsequent nitrogen deprivation increased ARA accumulation 2.5 and 1.7 times in the suspended and chitosan-immobilized microalgae, respectively. The study of the *Lobosphaera* sp. microbiome revealed that the immobilization of chitosan rather than the CTA exposure was the main factor displacing the taxonomic composition of the microbiome. The possibility and limitations of the use of chitosan-immobilized *Lobosphaera* sp. IPPAS C-2047 for HMP removal coupled with the production of valuable long-chain polyunsaturated fatty acids is discussed.

## 1. Introduction

The immobilization of microalgal cells on various synthetic and natural carriers attracted increasing interest in the field of wastewater treatment [1,2]. The main advantages of immobilized (attached) microalgal cultures are higher resistance to toxicants and overall robustness, increased rate of biomass, and value-added molecule accumulation as compared to suspended cultures [3]. Moreover, attached cultivation eliminates the need for costly cell-harvesting procedures such as centrifugation; attached cultures can be also used through several production cycles without a significant loss of their activity [1,4]. Immobilized microalgae cultivation was shown to be a promising technology for the removal of antibiotics from wastewater [3,5,6]. Microalgae can effectively remove antibiotics from wastewater (see [5,7] and references therein). For example, a high-rate algal pond (HRAP) system dominated by *Coelastrum* sp. removed 33 antibiotics (with an average concentration of 223 µg/L) from municipal wastewater with the average antibiotic removal rates being up to 50% higher than those of the conventional activated sludge process [8]. The immobilized microalgal biomass grown in suitable wastewater and enriched with nutrients, lipids, and carotenoids can be used as biofertilizers for the production of biofuel and value-added compounds [9].

The successful application of immobilized microalgae in wastewater treatment depends on the informed choice of the optimal carrier and immobilization technique [2]. Current progress in material science made available a broad spectrum of natural and synthetic materials for microalgae immobilization [1]. Among them, synthetic polymers (acrylamide resins, polyurethanes, polyvinyl alcohol, and polypropylene) feature high mechanical and chemical stability, but using of synthetic polymers entails the problem of the utilization of used cell carriers [2]. The key advantages of the natural carriers (loofa, sphagnum, turf, cellulose, carrageenan, and alginate) are hydrophilicity, biocompatibility, and safety for the environment should be balanced against their low porosity and mechanical stability in wastewater [2,4]. Particularly, the cross-linked chitosan-based polymeric materials as biocompatible, cost-effective, and non-toxic carriers are prospective for the immobilization of microalgae [2,10]. These polymers have a variety of potential applications as biosorbents for water biotreatment applications due to a high potential for sorption of heavy metal ions, dyes, phenol, and polychlorinated biphenyls [11].

A serious concern is that the leakage of antibiotics into the environment from wastewater treatment plants [5,12]. Most antibiotics are hardly removed by the bacteria of activated sewage sludge since the antibiotics are purposely designed to inhibit bacterial growth and metabolism [13]. Incomplete, slow degradation of the antibiotics leads to their increased persistence in the environment and enrichment of natural communities and wastewater treatment plants with pathogenic and opportunistic microorganisms that are resistant to antibiotics and are hence dangerous to human health [12,14]. Although concentrations of hazardous micropollutants including antibiotics currently encountered in waste streams are low in comparison with their total organics content, these micropollutants impose a serious threat to the environment due to their high biotoxicity. Although environmental concentrations of antibiotics are seldom reported to have acute toxicity to aquatic organisms, long-term negative effects on individual organisms and populations have been documented including the impact on microbial consortia [6].

As noted above, microalgae-based technology has been reported as an effective method to remove and degrade antibiotics with added benefits of CO_2_ fixation, accumulation of value-added products, nutrients, and other organic pollutants removal [6,15]. At the same time, little is known about the influence of antibiotics on the biology of immobilized microalgal cells and the efficiency of HMP removal from wastewater.

To the best of our knowledge, this work is the first test of the cross-linked chitosan-based polymer with immobilized microalgae for antibiotics removal from model wastewater. Along with studying the removal of a widespread antibiotic ceftriaxone (CTA), we studied, also for the first time, the influence of CTA on the accumulation of arachidonic acid (ARA) by suspended and immobilized *Lobosphaera* sp. Representatives of the genus *Lobosphaera* including the studied strain are the richest plant source of polyunsaturated ω-6 arachidonic acid (ARA) [16]. Arachidonic acid is an essential, structural, and functional constituent of cell membranes, it is used as a nutraceutical food and feed additive [17,18].

The results of this study would augment the development of the technologies with chitosan-immobilized microalgae intended for antibiotics removal from wastewater coupled with the production of valuable polyunsaturated fatty acids by attached microalgal cultures.

## 2. Results

**Figure 1:**
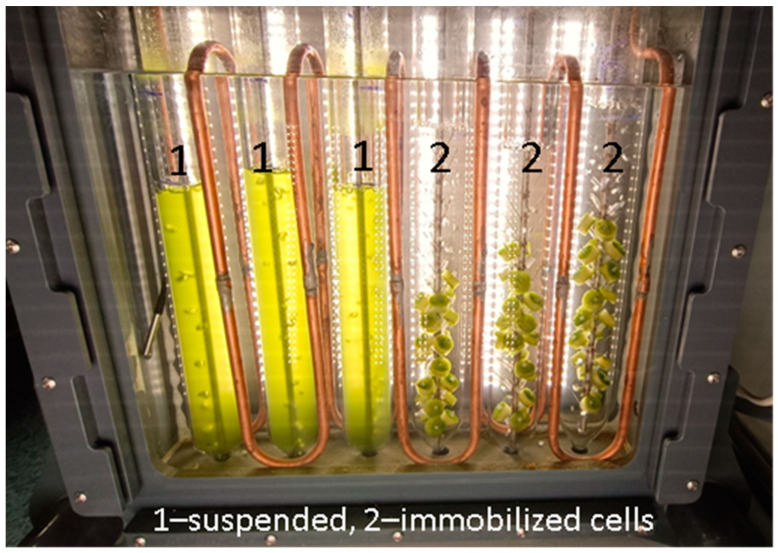
The suspended (1) and immobilized (2) cultures of *Lobosphaera incisa* IPPAS C-2047 incubated in the photobioreactor (see Methods).

Two types of experiments have been conducted in our study. The first type of experiment was carried out in flasks (see Methods) with suspended and immobilized *Lobosphaera* sp. with and without 20 mg/L of CTA. The aim of the flasks tests was to evaluate the interactive effects of CTA and the immobilization of chitosan carriers on the growth, photosynthetic activity, and microbiome of *Lobosphaera* sp. The second type of experiment was conducted in a bioreactor (Figure 1) with immobilized and suspended *Lobosphaera* sp in the presence of 20 mg/L of CTA to assess the effect of the immobilization and subsequent nitrogen deprivation on photosynthetic activity and the fatty acid profile of the cells with an emphasis on valuable ARA. Special attention was paid to the kinetics of CTA removal.

### 2.1. Accumulation of Chlorophyll by and Growth of the Cultures under N-Sufficient Conditions

**Figure 2:**
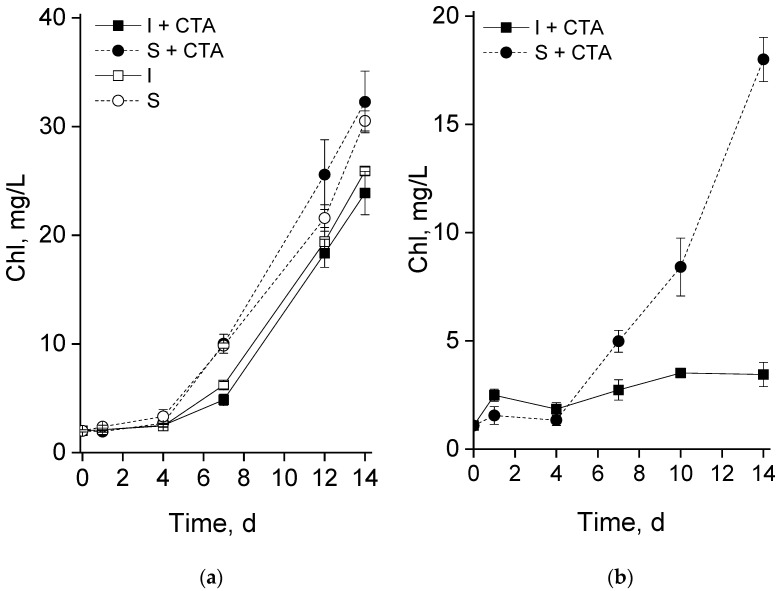
Changes in volumetric content of chlorophyll in the suspended (S) and immobilized (I) *Lobosphaera* sp. IPPAS C-2047 cultures during (**a**) pilot experiment in the flasks and (**b**) in the column photobioreactor during their incubation with (I+CTA or S+CTA, respectively) or without 20 mg/L ceftriaxone (I or S, respectively).

During the flask-based tests, both the suspended and the chitosan-immobilized cultures demonstrated, after a 3–4 d lag, a steady accumulation of chlorophyll indicative of culture growth (Figure 2a). The suspended culture accumulated chlorophyll faster than the immobilized one until the seventh day of the experiment but later the rates of chlorophyll accumulation by the cultures were commensurate with each other. The presence of CTA in the studied concentration did not exert a measurable effect on the culture growth in either suspended or immobilized culture.

Surprisingly, the cultures exhibited a drastically different behavior in the second type of experiment carried out in the glass column photobioreactor in the presence of CTA (Figure 2b). Thus, the kinetics of chlorophyll accumulation by the suspended culture was close to that documented in the flask-based tests (cf. the curves with closed squares in Figure 2a,b). By contrast, the chitosan-immobilized culture accumulated chlorophyll at a much slower rate than the suspended culture under the same experimental conditions or the immobilized culture incubated with CTA in flasks (cf. the curves with closed circles in Figure 2a,b). Notably, the decline showed by the immobilized cultures can hardly be explained by the effect of immobilization per se since it was not observed in our previous experiments with the same culture under the same experimental conditions (Figure S1).

### 2.2. Functioning of the Photosynthetic Apparatus of the Microalgal Cells

**Figure 3:**
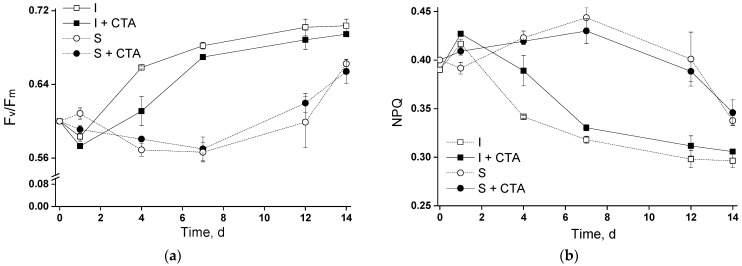
Changes in (**a**) potential maximal photochemical quantum yield of photosystem II (Fv/Fm) and (**b**) Stern-Volmer non-photochemical quenching of chlorophyll excitation in the suspended and immobilized *Lobosphaera* sp. IPPAS C-2047 cultures during pilot experiment in the flasks during their incubation with or without 20 mg/L ceftriaxone. For curve designations, see the legend to Figure 2.

**Figure 4:**
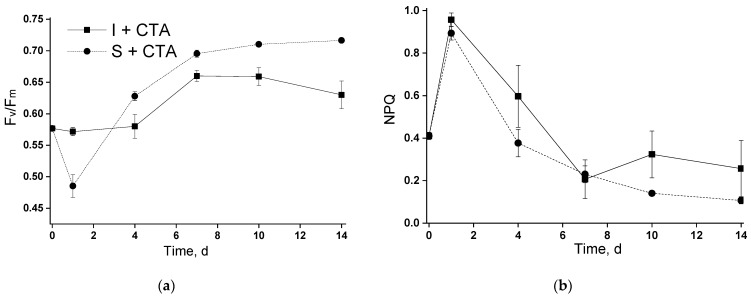
Changes in (**a**) potential maximal photochemical quantum yield of photosystem II (Fv/Fm) and (**b**) Stern–Volmer non-photochemical quenching of chlorophyll excitation in the suspended and immobilized *Lobosphaera* sp. IPPAS C-2047 cultures during the experiment in the column photobioreactor during their incubation with or without ceftriaxone. For curve designations, see the legend to Figure 2.

In view of the observed kinetics of chlorophyll accumulation, it was essential to compare the functioning of the photosynthetic apparatus in the experimental cultures (Figure 3 and Figure 4). During the flask tests, the cultures exhibited a characteristic pattern of changes in the magnitude of potential maximal photochemical quantum yield of photosystem II (Fv/Fm) which depended on the immobilization state and was only slightly affected by the presence of CTA (Figure 3a). Regardless of the cultivation conditions, the Fv/Fm did not change or even declined slightly during the first day after the beginning of the experiment remaining at the level of ca. 0.60, likely due to the mild stress caused by the culture handling during the inoculation of the flasks and/or cell carriers. Later, Fv/Fm in the immobilized cultures increased to 0.65–0.70, likely due to the accumulation of chlorophyll and an (Figure 2a) increase in cell self-shading resulting from it. Notably, the lag in Fv/Fm attributable to acclimation of the photosynthetic apparatus of the cells after inoculation was considerably longer in the suspended cultures (around eight days, circles in Figure 3a). This effect can be tentatively attributed to lower self-shading in the suspended culture and, hence, to higher effective per-cell light intensity in this case.

Expectedly, the magnitude of the NPQ parameter are indicative of the degree of engagement of the photoprotective mechanisms in the cells followed the opposite trend as compared with that of Fv/Fm (cf. Figure 3a,b). Thus, the initial decline in Fv/Fm corresponded to an increase in NPQ, suggesting that the thermal dissipation of the light energy absorbed in the excess under the stress condition was engaged by the cells under our experimental conditions. Later, after successful acclimation of the cells, relaxation of NPQ was observed. As with Fv/Fm, the immobilized cells showed a faster decline in NPQ than the suspended cells.

None of the parameters were profoundly affected by the presence of 20 mg/L CTA. The only exception comprises the immobilized culture around the fourth day of the experiment when the Fv/Fm of the immobilized culture with CTA was significantly lower (and NPQ was correspondingly higher) than that of the immobilized culture incubated without the antibiotic (cf. curves with squares in Figure 3a,b).

Similar trends of changes in the Fv/Fm and NPQ were observed in the bioreactor tests (in the cultures incubated in the photobioreactor; Figure 4). The synchronous spike of NPQ observed during the first 1–2 days of incubation in the photobioreactor of both suspended and immobilized cultures (Figure 4b) was likely associated with the acclimation of the microalgal cells to a different illumination condition but not with the presence of antibiotic (see below).

Overall, CTA in the studied concentration did not exert dramatic effects on the functioning of the photosynthetic apparatus of the studied strain of *Lobosphaera* sp. Notably, there was no sizeable effect of the antibiotic on the capacity of the cells to photochemically convert and/or thermally dissipate the absorbed light energy even in the case where severe retardation of chlorophyll accumulation took place (the immobilized culture incubated with CTA in the photobioreactor). 

### 2.3. Effects of the Immobilization and CTA on the Culture Microbiome

**Figure 5:**
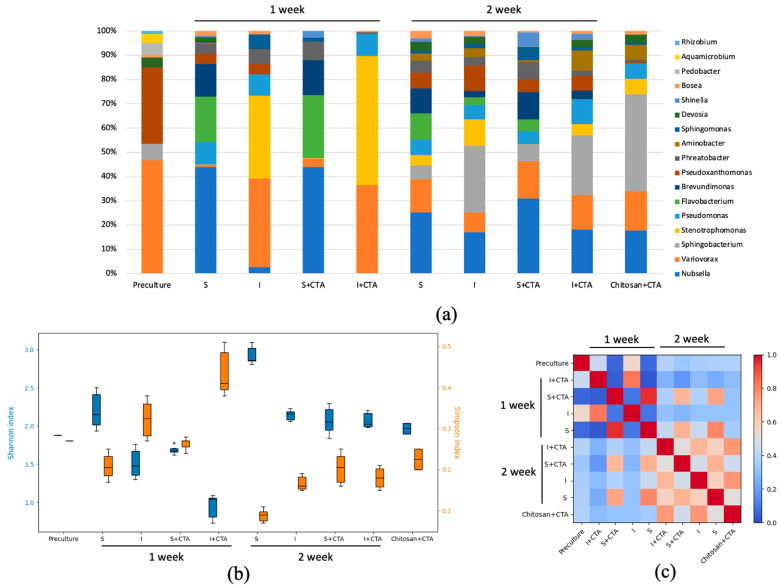
Metagenomic analysis of the suspended (S) and immobilized (I) *Lobosphaera* sp. IPPAS C-2047 culture with (S+CTA or I+CTA, respectively) or without (S or I, respectively) addition of 20 mg/L ceftriaxone, in comparison to the preculture and blank chitosan with CTA, at the end of the 1 and 2 week of incubation. (**a**) Genus level taxonomic structure; (**b**) α-diversity index of Shannon (blue) and Simpson (orange); (**c**) β-diversity index of Morishita.

The DNA metabarcoding data have been obtained only in the flask-based tests to reveal possible effects of the antibiotic treatment and the immobilization on the culture microbiome. The results revealed the presence of 120 genera of prokaryotic organisms in the cultures. Comparison of the taxonomic composition at the level of the most represented genera (Figure 5a) showed that the microbiomes of these samples changed significantly both in time (7 and 14 d) and depending on the treatment. At the end of the first week, the genus-level diversity of the sample was relatively narrow. By the end of the second week, the diversity of represented genera increased, but microbiomes of the same culture types (i.e., suspended or immobilized) were similar regarding the CTA presence. The microbial diversity of the cell-free samples of chitosan incubated with CTA did not change considerably during the two-week incubation.

The results of DNA metabarcoding were further analyzed in terms of biodiversity indices (Figure 5b). At the end of the first week of incubation, the lowest biodiversity was observed in the immobilized culture incubated with CTA, and the highest biodiversity of the microbiome was exhibited by the suspended cultures without the antibiotic, and the biodiversity of the suspended cultures with CTA was on an intermediate level. This trend largely remained by the end of the experiment.

Along with the assessment of inter-variant biodiversity, the uniformity of the distribution of taxonomic groups was assessed with Simpson’s index. The highest value of this index was found in the immobilized culture incubated with CTA for 7 d evident of the domination of certain groups in the microbiome. The lowest value of the Simpson’s index was observed in the suspended cultures with no CTA added after 14 d manifesting the even contributions of different taxonomic groups into the microbiome of these cultures.

Of special note are inverse trends of the Shannon–Weaver and Simpson indices during the experiment arising likely due to immobilization and/or the presence of CTA. The incubation with CTA resulted in a narrowing of the microbial diversity within the first week. It is possible to think that the microbiome in this case was overtaken by the organisms with resistance to CTA or those gaining a competitive advantage after displacement of the microbiome composition by the antibiotic. A lower biodiversity of the immobilized cultures’ microbiome may indicate that a selection of the microorganisms regarding their capacity of immobilization on the carrier takes place. One cannot exclude the potentiating effect of CTA due to its concentration by the carrier (see Section 2.4).

To visualize the degree of similarity of the culture microbiome in different experimental treatments, the Morishita β-diversity index values were calculated and visualized as a heat map (Figure 5c) showing three spectacular patterns. The first included the samples incubated for 7 d, where suspended and immobilized culture possessed peculiar microbiome compositions. The second pattern emerged from comparing of the samples obtained after 7 d and 14 d of cultivation, in which only suspended cultures possessed a similar microbiome composition regardless of CTA presence. The third pattern was represented by the increase in overall similarity of the microbiomes from different cultures regardless of immobilization and CTA.

### 2.4. Kinetics of Changes in the Residual CTA Concentration in the Medium

**Figure 6:**
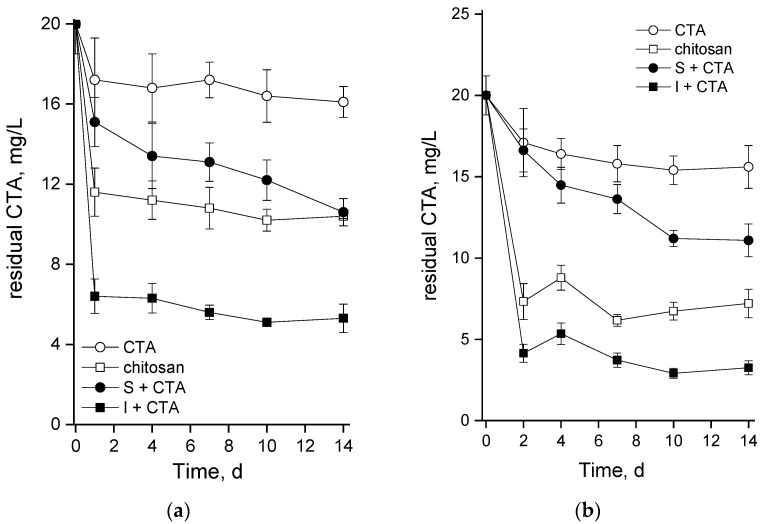
Changes in the residual concentration of ceftriaxone in the cell-free BG-11_M_ medium (CTA; open circles), cell-free BG-11_M_ medium with chitosan (chitosan; open squares), suspended (S+CTA), and immobilized (I+CTA) *Lobosphaera* sp. IPPAS C-2047 cultures during (**a**) experiments in the flasks and (**b**) in the column photobioreactor.

For a more confident interpretation of the results reflecting the microalgal culture condition, we monitored (by UPLC-ESI-MS) the actual changes of the antibiotic amount added to the medium at the beginning of the experiment (Figure 6). Since CTA is known to degrade in the presence of oxygen even without the participation of biological agents, a decline in its concentration in the medium-lacking cells and the chitosan carrier was monitored under conditions in which the cultures were incubated (light, bubbling, etc.). Regardless of the cultivation conditions, the abiotic degradation of CTA accounted for ca. 20% of the initially added amount of the antibiotic (see curves with open circles in Figure 6).

Our preliminary studies showed that CTA is readily adsorbed on the chitosan-based cell carriers. Therefore, the adsorption of CTA was separately monitored in un-inoculated flasks or columns containing the same amount of the carrier as was used in the experimental variant with the immobilized microalgae. Our estimations showed that the cell carrier itself can adsorb up to 45% or 60% of the added CTA during incubation of the flasks or in the columns of the bioreactor, respectively; see the curves with open squares in Figure 6a,b (these estimations also include abiotic oxidation of CTA). The most rapid decline of the CTA concentration in the medium took place during the first 1–2 d of the experiment, later, the trend of CTA removal from the medium slowed down or leveled off.

Obviously, abiotic oxidation and the adsorption on the carrier were the main processes contributing to the observed removal of CTA from the medium. A relatively small proportion of the removed CTA (several percent of the amount initially added to the culture) can be attributed to the microalgal cells. However, it is difficult to say was it due to the internalization of CTA with its subsequent metabolization or just from the adsorption on the cell surface. Indirect evidence of the predominance of the latter process can be seen in Figure 6: the suspended cultures which showed rapid growth (see Figure 2) also continued to remove CTA from the medium until the end of the observation period (closed circles in Figure 6a,b). The immobilized cultures showed a limited capacity for CTA removal after the initial rapid decline of the antibiotic concentration (closed squares in Figure 6a,b). Nevertheless, the bulk removal of CTA added at the concentration of 20 mg/L was, under our experimental conditions, as high as 65% (in the flasks) or 85% (in the bubbled columns).

### 2.5. Induction of Lipid Accumulation in Microalgal Cells by Their Nitrogen Deprivation

**Figure 7:**
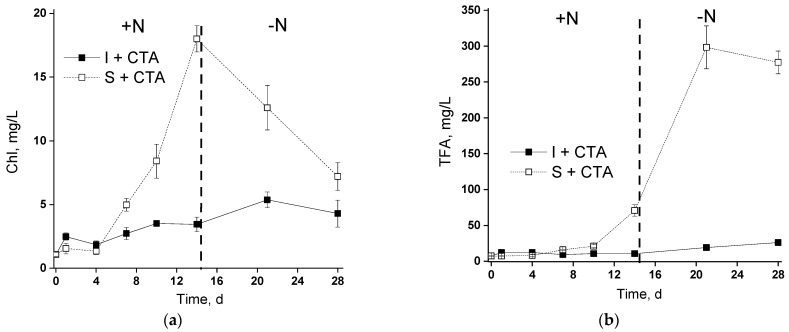

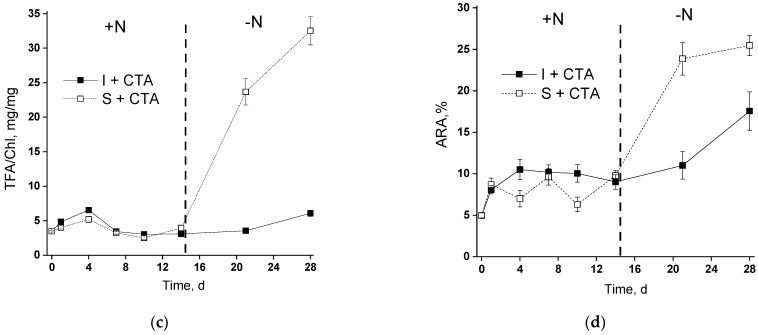
Induction of fatty acid accumulation in the cells of the suspended (S) and immobilized (I) *Lobosphaera* sp. IPPAS C-2047 cultures during their incubation in complete BG-11_M_ medium containing ceftriaxone (+N) and nitrogen-free, BG-11_M_–N medium that lacks the antibiotic (–N). The changes in (**a**) chlorophyll, (**b**) volumetric total fatty acid content, (**c**) total fatty acid per unit chlorophyll, and (**d**) arachidonic acid percentage of total fatty acids are shown.

The induction of lipid accumulation was accomplished by depriving the microalgal cells grown in the photobioreactor (see Figure 1), either suspended or immobilized on the chitosan-based carrier (Figure 7). The nitrogen starvation was induced by changing the complete BG-11_M_ medium with CTA for the nitrogen-free BG-11_M_ medium. The antibiotic was not added at the stage of nitrogen starvation since it can be a potential nitrogen source, complicating the interpretation of the results.

In the suspended culture, N deprivation induced a sharp decline in chlorophyll content, a typical response of microalgal cells to nitrogen deprivation (Figure 7a). Interestingly, the immobilized culture featuring a slow accumulation of chlorophyll under nitrogen-sufficient conditions continued to accumulate the pigment for ca. five days after nitrogen deprivation (closed squares in Figure 7a).

In the nitrogen-sufficient cultures, the changes in total fatty acids (TFA) of the cell lipids (Figure 7a,b) closely followed the dynamics of chlorophyll content. As a result, volumetric TFA content increased along with chlorophyll accumulation, but TFA expressed per unit as chlorophyll remained nearly constant in the N-sufficient cultures both in the immobilized and suspended N-sufficient cultures. Expectedly, the nitrogen deprivation triggered an increase in volumetric TFA and an even higher increase in TFA per unit as chlorophyll in the suspended culture. At the same time, only a modest increase in TFA was observed in the chitosan-immobilized microalgae. Neither nitrogen starvation nor the immobilization or CTA addition affected the qualitative profile of FA in the cell lipids of the *Lobosphaera* sp. (Tables S1 and S2).

The trend of changes in arachidonic acid (ARA) TFA percentage followed that of TFA (Figure 7b,c). Again, the increase in ARA percentage of TFA was more spectacular in the suspended culture than in the immobilized one (25% vs. 17%, respectively).

## 3. Discussion

Immobilization of the cells on biocompatible cell carriers is frequently employed in microalgal biotechnology to facilitate biomass harvesting as well as to increase the stress resilience of microalgae and hence the culture robustness [1,4]. Chitosan-based biopolymers are highly promising cell carriers for the immobilization of microalgal cells [11]. However, they can also be a source of nutrients interfering with the culture manipulation by controlled stress such as nitrogen deprivation for induction of lipids harboring valuable fatty acids. The use of attached cultivation and increased stress resilience ensued by is especially relevant for growing microalgae in waste- and sidestreams as a source of nutrients. Many types of wastewater (e.g., farming and municipal waste streams) contain sizeable amounts of hazardous micropollutants such as antibiotics [2,11,19]. At the same time, the information on possible effects of the presence of antibiotics on the biology of immobilized microalgal cells is scarce. In view of this, we attempted to reveal the interactive effects of the immobilization of the cells and a widespread antibiotic CTA on the production of a valuable polyunsaturated fatty acid ARA by its producer *Lobosphaera* sp. IPPAS C-2047. As a provocative test, we deliberately employed a high (20 mg/L) CTA concentration since it was shown that *Lobosphaera* sp. is tolerant of CTA in the environmentally relevant concentrations around 1–3 mg/L [20]. Therefore, the first type of experiment (flasks tests) has been dedicated to testing the potential acute toxicity of CTA to the selected microalgal strain and the effect of chitosan immobilization on the *Lobosphaera* sp. tolerance to the increased CTA concentration. Judging from the obtained results (Figure 2a and Figure 3), 20 mg/L CTA did not induce a profound deteriorative effect on the cells of *Lobosphaera* sp. cultivated in flasks regardless of the immobilization, only a slight inhibition of photosynthetic activity has been recorded in the immobilized cells at day four (Figure 3).

An unexpected result was represented by severe retardation of the growth (as manifested by chlorophyll accumulation) of the chitosan-immobilized culture in the presence of CTA. Remarkably, this effect was not accompanied by severe disorders of the photosynthetic apparatus (Figure 4). A plausible explanation can be related to an increase in the concentration of CTA due to its sorption on chitosan. This effect may be not so pronounced during incubation in flasks in the absence of vigorous mixing while in the bubbled columns, the rate of CTA adsorption (and hence the rate of the increase in its effective concentration in the vicinity of microalgal cells) was ca. 30% higher (open squares in Figure 6a,b). It seems that a sub-population of *Lobosphaera* sp. cells which was more resilient to increased CTA concentration and was effectively selected on the chitosan-based cell carrier. These cells might have retained a functional photosynthetic apparatus and the essential responses of their lipid metabolism to nitrogen deprivation manifested as an induction of lipid accumulation.

Taking into account the results of our previous experiments on the immobilization of *Lobosphaera* sp. on chitosan-based cell carriers one can conclude that the immobilization itself does not affect the FA profile (Tables S1 and S2), degree of lipid and, specifically, ARA induction profoundly (Figure S1, see also [19]). The typical ARA percentage of immobilized TFA was in the range of 20–25%. Therefore, the decline in the amount of TFA and ARA observed in the presence of CTA is attributable mostly to the effect of the antibiotic and not to the effect of the attachment to the carrier.

An intriguing question to be answered by this study was about the capacity of suspended and chitosan-immobilized cultures of green microalgae such as *Lobosphaera* sp. to remove CTA from the medium. Comparison of the abiotic degradation of CTA and its adsorption to the chitosan-based carrier with the bulk CTA removal (Figure 6) made it clear that the removal of CTA in our system occurred mainly due to these two processes and the contribution of the microalgal cells to CTA removal was minor. It is difficult to distinguish between the adsorption of CTA on the cell surface and uptake of the antibiotic by the cells. Still, one cannot rule out at least partial metabolization of CTA. Importantly, the cultures at the stage of nitrogen starvation were also affected by CTA despite replacement of the medium (see Methods) since a considerable amount of the antibiotic was adsorbed to the cell carrier (see Figure 6); likely, there was also some adsorption of CTA to the microalgal cell surface as well.

Diverse processes such as adsorption, advanced oxidation, and photocatalysis have been studied to remove antibiotics. As was summarized recently [5], the antibiotic removal efficiency by adsorption is highly adsorbent-dependent, and most of the adsorbents are expensive. Advanced oxidation and photocatalysis-based processes may be effective, but they need expensive catalysts; these processes can generate dangerous secondary pollutants. Advantages of the microalgae-based wastewater treatment process as a biological process are the absence of the need for expensive reagents and a low risk of secondary pollution [21]. Immobilization of the microalgal cells on biopolymer carriers increases the efficiency of the process, also due to the sorption capacity of the carrier itself. In our experiments, the CTA level was taken down to 6 mg/L or 4 mg/L.

To elucidate possible trends of changes in the *Lobosphaera* sp. culture microbiome, it was essential to evaluate the most represented microbial taxa and the effect of immobilization and CTA on them (Figure 5a). Notably, all the bacterial genera discovered are typical for microalgal cultures under different cultivation conditions. Many representatives of these genera belong to the PGPB (plant growth-promoting bacteria) group, such as *Variovorax*, *Stenotrophomonas*, *Shinella*, *Devosia*, *Sphingomonas*, etc. Representatives of microalgal parasites were not discovered. Importantly, some representatives of *Variovorax*, *Stenotrophomonas*, *Flavobacterium* are known to be resistant to a wide range of antibiotics, including CTA ceftriaxone, having the ability to degrade them [22,23].

Ample representation of the genera *Variovorax* and *Stenotrophomonas* in the chitosan-immobilized cultures can be due to the fact that the presence of the polymer creates favorable conditions for the development of these microorganisms. Thus, representatives of the genus *Variovorax* are known to be able to actively form biofilms, also on the surface of carriers [24]. Representatives of the genus *Stenotrophomonas* are known for the presence of chitinase and the ability to degrade chitosan [25]. At the same time, it was shown that some forms of chitosan also have antimicrobial activity, particularly suppressing the growth of certain representatives of the genus *Flavobacterium* [26].

The decline of the difference between the microbiome in different experimental treatments can be explained by (i) a decline in the CTA residual content and (ii) by acclimation and selection of the microorganisms favored by the presence of chitosan and CTA. This hypothesis is supported by the gradual proliferation of the microorganisms with modest resistance to ceftriaxone (*Pseudoxanthomonas*, *Devosia*, *Bosea*) in the samples containing CTA. The potential inhibitory effects of chitosan for certain bacteria (e.g., from the genus *Flavobacterium*) were also gradually overcome, likely via the formation of biofilm frequently observed on the surface of the carrier [19].

To conclude, the cultures of *Lobosphaera* sp., especially those immobilized on the chitosan-based cell carriers, turned to be suitable for removal of the antibiotics, especially taking into account that the CTA concentration in this study (20 mg/L) is higher than that typically encountered in the environment and even in the wastewater treatment plants. However, the attempts to couple the removal of hazardous pollutants such as antibiotics with the production of valuable microalgal metabolites should use caution since toxic effects can emerge unexpectedly due to the concentration of the pollutants on the carrier. One should also be aware of the possible enrichment of the microbiome of the attached microalgal cultures with antibiotic-resistant bacteria.

## 4. Materials and Methods

### 4.1. Synthesis of the Cross-Linked Chitosan-Based Polymers

The cross-linked chitosan polymers were synthesized in the Laboratory of Polymer Materials of the Kurchatov Institute Research Center. Two mg of chitosan ChitoClear HQG 800 (Primex, Island; molecular weight of 600 kDa) were dissolved in 98 mL of 2% aqueous solution of acetic acid (LLC “Component-Reactiv”, Tambov, Russia). The mixture was homogenized using a magnetic stirrer (Heidolph MR Hei-Tec, Schwabach, Germany) for 24 h at 23 °C. The chitosan-based carrier polymers were synthesized from the solution of chitosan and glutaraldehyde as cross-linking agents using lyophilization technique. An aliquot of 0.76 mL of 2.5% aqueous solution of glutaraldehyde was added to the chitosan mixture. After 15 min stirring with a magnetic stirrer, the mixture was placed in 12-well plates (2.7 mL per well), incubated in a freezer (24 h at −25 °C), and lyophilized using ALPHA 1-4LSC lyophilizer (Martin Christ, Germany) at 0.250 mbar for 24 h [27].

The resulting carriers in the form of porous discs (14 mm diameter, 10 mm thickness) were used in the experiments.

### 4.2. Strain and Cultivation Conditions

*Lobosphaera* sp. IPPAS C-2047 used in the presented investigation as the model microalga was obtained from the Culture Collection of K.A. Timiryazev Institute of Plant Physiology (IPPAS, Russian Academy of Science).

Preculture of *Lobosphaera* sp. IPPAS C-2047 was grown at 20 °C or 23 °C in a shaker incubator (New Brunswick, Innova-44R, Enfield, CT, USA) at 120 rpm in 0.75 L flasks with 0.3 L of P-enriched modified BG-11 [28] medium, BG-11_M_ (g/L: NaNO_3_ = 0.74, KNO_3_ = 0.9, K_2_HPO_4_ = 0.181, KH_2_PO_4_ = 0.089, MgSO_4_·7H_2_O = 0.075, CaCl_2_·2H_2_O = 0.036, citric acid = 0.006, ferric ammonium citrate = 0.006, Na_2_EDTA·2H_2_O = 0.001, Na_2_CO_3_ = 0.02, BG–11 trace metal solution) at 40 μmol PAR (photons/m^2^/s) provided by daylight fluorescent tubes (Philips TL-D 36W/54-765) as measured with a LiCor 850 quantum sensor (LiCor, Lincoln, NE, USA) and the atmospheric CO_2_ level. Culture pH was measured aseptically with a bench-top pH-meter (Hanna Instruments, Ann Arbor MI, USA).

To obtain the suspended cultures for the first type of the experiment (flasks tests), equal aliquots of the *Lobosphaera* sp. pre-culture were pelleted by centrifugation (5 min, 1000× *g*) and resuspended to ca. 1.5 mg chlorophyll/L in BG-11_M_ medium or in the same medium supplemented with CTA (LLC “Biosintez,” Penza, Russia) to the final concentration of 20 mg/L and placed in 100 mL vented cultivation flasks (TPP, Trasadingen, Switzerland). The experimental CTA concentration was chosen based on the results of preliminary experiments. Since CTA at concentrations below 10 mg/L were rapidly and completely adsorbed by the chitosan carrier and CTA in concentrations below 5 mg/L, it did not exert detectable effects on the *Lobosphaera* sp. within 10–14 d, a concentration of 20 mg/L was chosen.

In the second type of experiment, (bioreactor tests), the microalgal cell suspension was placed into the glass columns (600 mL; 4 cm i.d.; Figure 1), containing 400 mL of the cell suspension. The columns were incubated in a temperature-controlled water bath at 27 °C with constant bubbling with air passed through a 0.22 μm bacterial filter (Merck-Millipore, Billerica, MA, USA) and delivered at a rate of 300 mL/min (STP) at 40 μmol PAR (photons/m^2^/s).

Immobilization of the cells has been carried out as follows. A group of the pre-culture aliquots was gently re-suspended in BG-11_M_ and pipetted evenly on the surface of the chitosan discs to carry out the immobilization of the cells (the final concentration of chlorophyll was ca. 24 µg/disc). For the pilot experiment, the chitosan discs with firmly attached *Lobosphaera* sp. cells were placed into the 100 mL vented-cap cultivation flasks (TPP, Switzerland) containing BG-11_M_ medium supplemented with 20 mg/L of CTA or BG-11_M_ lacking the antibiotic. For the bioreactor tests, the chitosan discs with attached *Lobosphaera* sp. cells were fixed on the in-house made stainless steel spikes within the bioreactor column (Figure 1) containing BG-11_M_ medium supplemented with CTA to the final concentration of 20 mg/L.

In the first type of the experiment, the flasks with the suspended or immobilized *Lobosphaera* sp. cells were cultured for 14 days on the Innova-44R incubator shaker at 120 rpm under the condition specified above. In the second type of the experiment, the columns were incubated in the bioreactor under the conditions described above.

To induce nitrogen starvation for lipid accumulation, the complete BG-11_M_ medium in the columns of the photobioreactor was replaced with a nitrogen-free medium (BG-11 _M_–N) after 14 d of cultivation. Specifically, the suspended cells were gently harvested by centrifugation (2 min, 1000× *g*), washed with BG-11_M_–N medium, and re-suspended in the same medium. The immobilized cultures were transferred into identical columns with BG-11_M_–N medium. All the cultures were incubated in the photobioreactor for another 14 days at 200 μmol PAR (photons/m^2^/s); all the other conditions were as specified above.

### 4.3. Chlorophyll and Nutrients Assay

Growth estimation was based on the chlorophyll content measurements. For chlorophyll assay determination, an aliquot of the cell suspension was sampled and harvested by centrifugation for 5 min at 3000 g. Total Chl was extracted by heating the cell pellet with 2 mL of dimethyl sulfoxide (DMSO) for 10 min at 70 °C. From the cells attached to the carrier, chlorophyll was extracted by adding 2 mL of DMSO to the carrier with the cells and heating for 10 min at 70 °C. Concentration of chlorophyll was determined in the DMSO extracts with an Agilent Cary 300 spectrophotometer (Walnut Creek, CA, USA) using equations reported in [29].

The residual phosphate and nitrate contents were checked using Thermo Dionex ICS 1600 HPLC (Sunnyvale, CA, USA) with a conductivity detector and IonPac AS12A anionic analytical column (5 μm; 2 × 50 mm). The column temperature was maintained at 30 °C. The ions were eluted isocratically with 2.7 mM sodium carbonate/0.3 mM sodium bicarbonate buffer (flow rate of 0.3 mL/min).

### 4.4. Photosynthetic Activity and Photoprotective Mechanism Probing

Viability of the immobilized and suspended *Lobosphaera* sp. cells was evaluated via their photosynthetic activity with a FluorCam FC 800-C (PSI, Drasov, Czech Republic) kinetic fluorescence imager. The chitosan carriers with immobilized cells or 5 mL aliquots of suspended culture were transferred into well plates and sealed aseptically. Estimations of the photosynthetic activity of the microalgal cells were obtained by recording Chl *a* fluorescence induction curves. The recorded curves were processed by the built-in software of the fluorometer, and the following parameters indicative of the functional condition of the photosynthetic apparatus of the microalgal cells were calculated: potential maximum photochemical quantum yield of photosystem II, Q*_y_* = (Fm − Fo)/Fm = Fv/Fm, and non–photochemical quenching of the electron excitation energy in the light-harvesting antenna (parameter NPQ, NPQ = Fm/Fm’ − 1) where Fo and Fm are the minimum and the maximum fluorescence of chlorophyll *a* in dark-adapted cells recorded in microalgal cells illuminated with a weak measuring light and a saturating pulse of white light, respectively; Fm’—maximum fluorescence intensity recorded in microalgal cells illuminated by actinic, i.e., light driving photosynthesis [30]. The PAM measurements were carried out after 10 min of dark adaptation. Chlorophyll fluorescence was excited at 650 nm and recorded in the red region of the spectrum (λ > 680 nm). After the measurements, the samples were returned to the corresponding cultivation flacks or the bioreactor column under aseptic conditions. This procedure allowed repeated assessments of the attached and suspended cells under our experimental conditions.

### 4.5. Assay of Cell Lipid Fatty Acid Composition

Total cell lipids were extracted from these samples according to Folch [31]: the cell pellets or the cells on the carrier were homogenized in a mixture of chloroform and methanol (2:1, by volume). Distilled water was added to the homogenate in the amount of 20% of the homogenate volume, the mixture was dark incubated overnight at 4 °C, and centrifuged (10 min, 3000 g) until phase separation. The chloroform phase of the extract was evaporated to dryness on a Heidolph Laborota 4000 rotary evaporator (Heidolph, Schwabach, Germany) at 30 °C. As an internal standard, 50 micrograms of margaric acid (C17:0) were added to the samples. The samples were transmethylated by incubation with 2% sulfuric acid in methanol for 1.5 h at 80 °C. Fatty acid (FA) methyl esters were extracted with 2 mL of n-hexane. In the case of immobilized cells, the carriers with the cells were ground and extracted as described above.

The fatty acid (FA) profile of total cell lipids was analyzed using an Agilent 7890A gas chromatograph (Agilent Technologies, Santa Clara, CA, USA) equipped with a 30 m HP-5MS UI capillary column (30 m × 0.25 mm × 0.25 microns; Agilent, USA). Helium with a flow rate of 1 mL/min was used as a carrier gas (for more details, see [32]).

The proportion of individual FA in the total FA of the *Lobosphaera* sp. cell lipids was inferred from the corresponding peak area. The absolute FA contents were calculated relative to the internal standard (C17:0) peak area.

### 4.6. UPLC-ESI-MS Assay of Ceftriaxone

The lyophilized culture broth samples were dissolved in methanol–water mixture (1:1, by vol.) and centrifuged (15 min, 15,000 rpm; centrifuge ELMI CM-50, Latvia). The samples were chromatographed on an ACQUITY UPLC H-Class PLUS (Waters, Santa Clara, CA, USA) equipped with a time-of-flight mass-selective detector Xevo G2-XS TOF (Waters, USA). An 0.5 µL sample aliquot was injected on a Titan C18 (100 × 2.1 mm, 1.9 µm; Supelco, Bellefonte, CA, USA) column maintained at 50°C and eluted with a gradient of 10 mM solution of ammonium acetate in deionized (Simplicity UV, Millipore, France) water (solvent A) and LC-MS grade (Panreac, Barcelona, Spain) acetonitrile (solvent B) delivered at a rate of 0.5 mL/min. The following gradient program was used (vol. % of the solvent B): 0–1 min, from 5% to 15%; 1–2 min, from 15% to 65%; 2–3 min, from 65% to 85%; 3–5 min, from 85% to 95% (see also [33]). The analysis was carried out in the negative ion detection mode (*m*/*z* range of 100–1900), the ion source parameters were: ion source temperature—150 °C, desolvation temperature—650 °C; capillary voltage—3.0 kV; sample injection cone voltage—30 V; nitrogen (desolvation gas) flow rate—1101 L/h. The recorded data were processed with MassLynx v. 4.2 software (Waters, USA) and CTA was quantified using external calibration method.

### 4.7. Taxonomical Profiling of the Culture Microbiome

Cell pellet was collected from 2–5 Ml of each freshly taken sample by centrifugation (8000× *g*) for 10 min and immediately frozen at −80 °C and stored until analysis. For metagenomic DNA (mgDNA) extraction, the cell pellets were frozen in liquid nitrogen and ground to a fine powder using homogenizing pestles (SSIBio Corp., Lodi, CA, USA) in a 1.5 Ml microcentrifuge tube. The freezing-homogenization procedure was repeated three times for each sample. MgDNA was isolated from the obtained homogenates using the Dneasy Plant Pro kit (QIAGEN, Hilden, Germany) according to the manufacturer’s instructions, replacing the sample mixing procedure on the Vortex with manual mixing by shaking the tube.

Amplification of the 16S Rrna gene fragment with the V4 hypervariable region and preparation of libraries for sequencing was carried out as described previously [34] using oligonucleotide primers F515 (5′-gtgccagcmgccgcggtaa-3′) and R806 (5′-ggactacvsgggtatctaat-3′) [35]. Sequencing was performed on a MiSeq instrument (Illumina, San Diego, CA, USA) using a MiSeq Reagent Kit v3 (600-cycle) (part number MS-102-3003, Illumina, USA) for paired-end read (2 × 300 bp). Initial data processing—sample demultiplexing and removal of adapters—was carried out using the Illumina v1.8.46 software (Illumina, USA). Further denoising, sequence merging, restoration of original phylotypes (ASV, Amplicon sequence variant), deletion of chimeric reads, and taxonomic classification of the resulting ASVs were performed in the R v. 4.3.1 software environment using the dada2 [36], phyloseq [37], and DECIPHER [38] packages, as well as the SSU 16s rRNA SILVA database (release 132) [39]. When analyzing the results of DNA metabarcoding, unclassified readings were removed from the samples, as well as those taxonomic groups that corresponded to one unpaired reading. For the number of readings, the average value was calculated for two biological replicates, for each of which two technical replicates were made. The Shannon–Weaver a-diversity (H) and Morishita β-diversity indices were calculated [40]. Morishita index values were visualized using an algorithm in the Python programming language (version 3.7.1) using the Matplotlib library.

### 4.8. Statistical Treatment

Under the specified conditions, three independent experiments were carried out for each treatment repeated in triplicate. The average values (*n* = 9) and corresponding standard deviation are shown unless stated otherwise. The significance of difference if the average values were analyzed using Student’s *t* tests.

## Data Availability

The data are available from the corresponding author upon a reasonable request.

## Supplementary Materials

The following supporting information can be downloaded at https://www.mdpi.com/article/10.3390/ijms241310988/s1.
