# Supplementary material for: Interactive Effects of Ceftriaxone and Chitosan Immobilization on the Production of Arachidonic Acid by and the Microbiome of the Chlorophyte Lobosphaera sp. IPPAS C-2047
Source: Int J Mol Sci. 2023 Jul 1;24(13):10988. doi: 10.3390/ijms241310988 (PMC10341515; doi:10.3390/ijms241310988)
Supplement: Supplementary file 1 [file ijms-24-10988-s001.zip › ijms-2450097-supplementary.pdf]

Online supplementary to the article:

Interactive effects of ceftriaxone and chitosan immobilization on the production of arachidonic acid by and the microbiome of the chlorophyte Lobosphaera sp. IPPAS C-2047

Svetlana Vasilieva [1,2], Alexandr Lukyanov [1], Christina Antipova [3], Timofei Grigoriev [3], Elena Lobakova [1,2], Olga Chivkunova [1], Pavel Scherbakov [1], Petr Zaytsev [1,2], Olga Gorelova [1], Tatiana Fedorenko [1], Dmitry Kochkin [1,4] and Alexei Solovchenko [1,2,*]

[1] Faculty of Biology, Lomonosov Moscow State University, 1-12 Leninskie Gory, 119234 Moscow, Russia; vankat2009@mail.ru (S.V.); elena.lobakova@gmail.com (E.L.); olga.chivkunova@mail.ru (O.C.); cyano@mail.ru (P.S.); zaytsevpa@my.msu.ru (P.Z.); ogo439@mail.ru (O.G.); tatfed@mail.ru (T.F.); dmitry-kochkin@mail.ru (D.K.); solovchenkoae@my.msu.ru (A.S.)

[2] Institute of Natural Sciences, Derzhavin Tambov State University, Komsomolskaya Square 5, 392008 Tambov, Russia

[3] Laboratory of Polymeric Materials, National Research Center "Kurchatov Institute", 123098 Moscow, Russia; kris444ti@yandex.ru (C.A.), timgrigo@yandex.ru (T.G.)

[4] Timiryazev Institute of Plant Physiology, Russian Academy of Sciences, Botanicheskaya St. 35, 127276 Moscow, Russia

* Correspondence: solovchenkoae@my.msu.ru; Tel.: +7-(495)-939-35-87

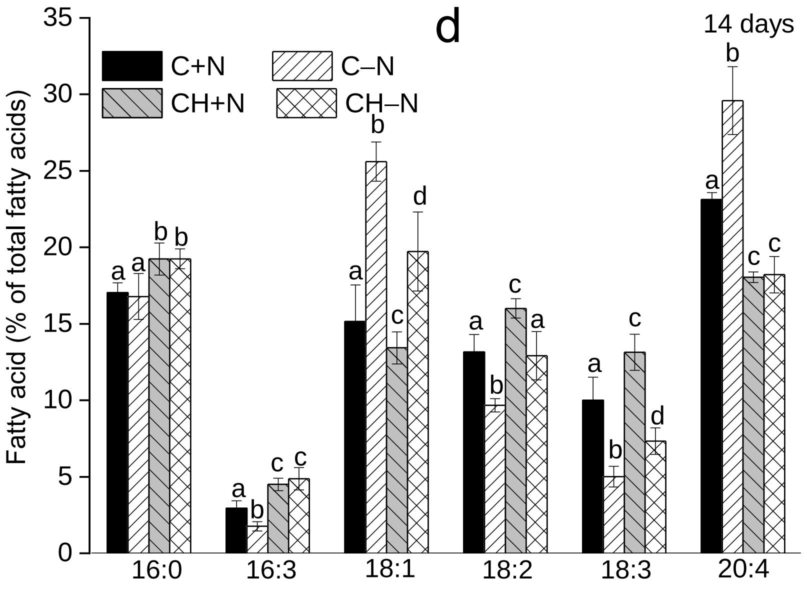

Figure S1. Figure 3d reprinted from [19] demonstrating that the accumulation of arachidonic acid in the immobilized and suspended cultures *Lobosphaera* sp. IPPAS C-2047 is similar ranging from 17 to 30% of the total fatty acids.

The profiles of the most abundant FAs among the total lipids in the cells of *Lobosphaera* sp. IPPAS C-2047 suspended (C+N, C–N) and immobilized (CH+N, CH–N) on chitosan in complete (C+N, CH+N) or N-free (C–N, CH–N) BG11$_M$ medium for 14 days are presented. Data are presented as means ± STD (n = 9). Lowercase letters indicate significant differences between experimental variants at $p < 0.05$

Table S1. Fatty acid profile of the suspended cells of *Lobosphaera* sp. IPPAS C-2047 during their incubation in complete and nitrogen-free BG-11M medium.

**FA, % of TFA**	**+ N**						**– N**	
	**– CTA** [1]			**+ CTA**			**– CTA**	
**Incubation time**	**0 d**	**1 d**	**4 d**	**7 d**	**10 d**	**14 d**	**21 d**	**28 d**
C14:0	3.7 ± 0.25	2.1 ± 0.9	2.3 ± 0.5	0.9 ± 0.3	2.2 ± 0.3	1.6 ± 0.3	0.6 ± 0.1	0.6 ± 0.1
C14:1Δ7	0.9 ± 0.1	0.8 ± 0.3	0.7 ± 0.3	0.4 ± 0.1	0.6 ± 0.2	0.3 ± 0.1	0.1 ± 0.1	0.1 ± 0.1
C15:0	2.9 ± 0.15	2.9 ± 1.1	2.9 ± 0.5	2.5 ± 0.3	6.2 ± 0.5	1.9 ± 0.2	0.4 ± 0.1	0.4 ± 0.1
C15:1Δ7	1.8 ± 0.11	1.8 ± 0.3	1.5 ± 0.4	1.9 ± 0.2	1.8 ± 0.3	0.9 ± 0.2	0.2 ± 0.1	0.1 ± 0.1
**C16:0**	**31.8 ± 2.4**	**31.6 ± 3.2**	**30.7 ± 4.1**	**25 ± 3.1**	**29.5 ± 1.6**	**23.9 ± 1.6**	**15.5 ± 1.1**	**18.3 ± 2.7**
C16:1Δ7	2.4 ± 0.3	5.1 ± 0.9	4.6 ± 1.1	4.7 ± 0.3	5.9 ± 0.7	3.3 ± 0.7	0.3 ± 0.1	0.6 ± 0.2
C16:1Δ9	3.3 ± 0.4	N/D	0.7 ± 0.3	0.2 ± 0	N/D	N/D	0.4 ± 0.1	0.4 ± 0.1
C16:2Δ7,10	1.3 ± 0.1	1.7 ± 0.3	1.2 ± 0.1	1.6 ± 0.3	2.7 ± 0.2	3.8 ± 0.2	0.6 ± 0.1	0.5 ± 0.2
C16:3Δ7,10,13	2.1 ± 0.2	1.5 ± 0.6	1.4 ± 0.3	3.3 ± 0.4	2.8 ± 0.3	3.9 ± 0.6	1.4 ± 0.1	0.9 ± 0.3
C16:4Δ4,7,10,13	1.1 ± 0	1.2 ± 0.4	1.9 ± 0.3	2.5 ± 0.5	4.5 ± 1	3.7 ± 0.4	0.2 ± 0.1	0.1 ± 0.1
C18:0	7.4 ± 1.2	4.6 ± 1.3	3.6 ± 0.6	2.7 ± 0.4	3.6 ± 0.6	2.2 ± 0.7	1.1 ± 0.1	1.8 ± 0.5
**C18:1Δ9**	**19.2 ± 2.1**	**17.1 ± 2.4**	**22.7 ± 2.5**	**22 ± 3.2**	**16.6 ± 2.6**	**20.1 ± 2.6**	**35.5 ± 1.5**	**35.6 ± 2.4**
C18:1Δ11	4.5 ± 0.5	5.1 ± 0.7	4.6 ± 0.3	3.3 ± 0.2	2.3 ± 0.2	2.3 ± 0.2	N/D	N/D
**C18:2Δ9,12**	**8.3 ± 0.7**	**8.8 ± 1.4**	**8 ± 1.2**	**9.6 ± 1.3**	**8.6 ± 1.1**	**12.8 ± 2.9**	**13.1 ± 0.3**	**10.1 ± 1.2**
C18:3Δ6,9,12	0.8 ± 0.1	0.6 ± 0.2	0.6 ± 0.2	1.5 ± 0.4	0.6 ± 0.2	0.6 ± 0.2	0.8 ± 0.1	0.5 ± 0.3
C18:3Δ9,12,15	2.9 ± 0.2	4.1 ± 1.2	2.6 ± 0.3	3.8 ± 0.6	2.9 ± 0.1	6.2 ± 0.9	3 ± 0.1	2.1 ± 0.4
C20:1Δ11	0.4 ± 0.1	0.6 ± 0.1	0.9 ± 0.1	1.6 ± 0.1	0.6 ± 0.2	0.4 ± 0.1	0.3 ± 0.1	0.2 ± 0.1
C21:0	N/D	0.2 ± 0.1	0.5 ± 0.1	0.5 ± 0.1	0.4 ± 0.1	0.2 ± 0.1	0.2 ± 0.1	0.2 ± 0.1
C20:2Δ11,14	N/D	0.4 ± 0.2	0.3 ± 0.1	0.9 ± 0.3	0.8 ± 0.2	0.7 ± 0.2	0.1 ± 0.1	0.1 ± 0.1
C20:3Δ8,11,14	N/D	0.3 ± 0.1	0.4 ± 0.2	0.6 ± 0.2	0.6 ± 0.1	0.6 ± 0.3	1.2 ± 0.2	1.1 ± 0.1
**C20:4Δ5,8,11,14**	**5 ± 0.3**	**8.7 ± 0.8**	**7.1 ± 1**	**9.6 ± 1**	**6.3 ± 0.9**	**9.8 ± 0.6**	**23.8 ± 2**	**25.5 ± 1.2**
C20:5Δ5,8,11,14,17	0.3 ± 0	0.8 ± 0.1	0.8 ± 0.3	0.9 ± 0.2	0.5 ± 0.1	0.8 ± 0.2	1.2 ± 0.1	0.8 ± 0.1

[1] – CTA—no ceftrixaone added; + CTA —20 mg/L ceftriaxone added.

Table S2. Fatty acid profile of the cells of *Lobosphaera* sp. IPPAS C-2047 immobilized on the chitosan-based cell carrier during their incubation in complete and nitrogen-free BG-11M$_0$ medium.

**FA, % of TFA**	**+ N**						**– N**	
	**– CTA** [1]			**+ CTA**			**– CTA**	
**Incubation time**	**0 d**	**1 d**	**4 d**	**7 d**	**10 d**	**14 d**	**21 d**	**28 d**
C14:0	3.7 ± 0.25	2.8 ± 0.3	2.4 ± 0.4	2.3 ± 0.3	2.2 ± 0.2	1.7 ± 0.2	1.8 ± 0.3	1.9 ± 0.2
C14:1Δ7	0.9 ± 0.1	0.6 ± 0	0.5 ± 0.1	0.5 ± 0.2	0.5 ± 0.2	0.4 ± 0.2	0.3 ± 0.1	0.7 ± 0.4
C15:0	2.9 ± 0.15	2.5 ± 0.1	2.9 ± 0.2	3.4 ± 0.1	3.1 ± 0.3	2.7 ± 0.3	2.1 ± 0.3	2.4 ± 0.5
C15:1Δ7	1.8 ± 0.11	0.8 ± 0.3	0.9 ± 0.4	2.2 ± 0.1	2.1 ± 0.5	2.1 ± 0.5	1.2 ± 0.2	1.5 ± 0.2
**C16:0**	**31.8 ± 2.4**	**32.1 ± 2.1**	**28.2 ± 1.8**	**29.3 ± 2.2**	**29.6 ± 1.4**	**29.6 ± 1.4**	**25.7 ± 1.6**	**23.0 ± 3.6**
C16:1Δ7	2.4 ± 0.3	2.4 ± 0.3	3.9 ± 2.7	2.6 ± 0.3	2.6 ± 0.6	2.2 ± 0.6	2 ± 0.6	2.4 ± 1.1
C16:1Δ9	3.3 ± 0.4	3 ± 0.1	1.9 ± 1.7	3.8 ± 0.3	3.1 ± 0.1	3.2 ± 0.1	2.2 ± 0.1	2.6 ± 0.3
C16:2Δ7,10	1.3 ± 0.1	1.8 ± 0.1	1.4 ± 0.3	1.5 ± 0.1	2.2 ± 0.1	2 ± 0.1	2.1 ± 0.3	1.9 ± 0.2
C16:3Δ7,10,13	2.1 ± 0.2	2.3 ± 0.1	2.5 ± 0.4	2.4 ± 0.6	3.8 ± 0.6	3.3 ± 0.6	2.5 ± 0.3	2.3 ± 0.9
C16:4Δ4,7,10,13	1.1 ± 0	0.7 ± 0.1	1 ± 0.3	1.3 ± 0.1	1.6 ± 0.2	1.1 ± 0.2	3.2 ± 1.1	2.3 ± 0.2
C18:0	7.4 ± 1.2	4.4 ± 0.6	3.8 ± 0.3	2.9 ± 1.1	2.6 ± 0.5	3.3 ± 0.5	2.9 ± 0.7	3.1 ± 0.5
**C18:1Δ9**	19.2 ± 2.1	14.3 ± 0.4	16.7 ± 1.6	15.1 ± 2.3	12.2 ± 2.2	13.4 ± 2.2	18.1 ± 3.3	14.9 ± 1.5
C18:1Δ11	4.5 ± 0.5	5 ± 0.4	3.9 ± 0.1	3.2 ± 0.4	3.3 ± 0.8	3.3 ± 0.8	6.2 ± 0.8	5.2 ± 0.3
**C18:2Δ9,12**	**8.3 ± 0.7**	**9 ± 0.6**	**9 ± 0.9**	**8.1 ± 1.4**	**9.8 ± 0.5**	**10.9 ± 0.5**	**10.5 ± 0.7**	**10.3 ± 1.9**
C18:3Δ6,9,12	0.8 ± 0.1	0.5 ± 0	0.5 ± 0.1	0.9 ± 0.9	0.4 ± 0.1	1 ± 0.1	0.3 ± 0.1	0.3 ± 0.1
C18:3Δ9,12,15	2.9 ± 0.2	6.9 ± 0.5	6.9 ± 1.1	6.6 ± 1.1	8.1 ± 1.5	7.5 ± 1.5	5.4 ± 0.5	5.2 ± 1.2
C20:1Δ11	0.4 ± 0.1	0.9 ± 0.4	0.7 ± 0.2	1.1 ± 0.9	0.6 ± 0.2	1.2 ± 0.2	0.4 ± 0.2	0.4 ± 0.1
C21:0	N/D	0.3 ± 0.1	0.1 ± 0.1	0.3 ± 0.2	0.2 ± 0.1	0.1 ± 0.1	0.1 ± 0.1	0.1 ± 0.1
C20:2Δ11,14	N/D	0.3 ± 0.2	0.2 ± 0.1	0.3 ± 0.1	0.2 ± 0.1	0.1 ± 0.1	0.3 ± 0.2	0.1 ± 0.2
C20:3Δ8,11,14	N/D	0.4 ± 0.3	0.8 ± 0.1	0.9 ± 0.2	0.6 ± 0.1	0.9 ± 0.1	0.5 ± 0.1	0.6 ± 0.2
**C20:4Δ5,8,11,14**	**5 ± 0.3**	**8.1 ± 0.5**	**10.6 ± 1.2**	**10.2 ± 1.1**	**10.1 ± 1.7**	**9 ± 0.9**	**11 ± 1.7**	**17.5 ± 2.3**
C20:5Δ5,8,11,14,17	0.3 ± 0	1 ± 0.2	1.3 ± 0.3	1.4 ± 0.4	1.1 ± 0.4	1 ± 0.4	1.2 ± 0.1	1.1 ± 0.1

[1] – CTA—no ceftrixaone added; + CTA —20 mg/L ceftriaxone added.
